# Synaptic transmission promotes brain metastatic outgrowth in breast cancer

**DOI:** 10.1172/jci.insight.193119

**Published:** 2025-09-02

**Authors:** Jayanta Mondal, Patrick Nylund, Prit Benny Malgulwar, William E. Johnson, Jason T. Huse

**Affiliations:** 1 Department of Translational Molecular Pathology,; 2 The MDACC/UTHealth Graduate School of Biomedical Sciences, and; 3 Department of Pathology, University of Texas MD Anderson Cancer Center (MDACC), Houston, Texas, USA.

**Keywords:** Neuroscience, Oncology, Breast cancer, Synapses, Therapeutics

## Abstract

This work demonstrates that normal neuron-to-neuron signaling machinery is hijacked by metastasizing cancer cells during their outgrowth in the central nervous system.

**To the Editor:** Brain metastases (BMs) develop in 20%–40% of patients with breast cancer (1), and patients with triple-negative breast cancer (TNBC) demonstrate particularly high incidence (28%) and poor median survival (3.7 months) (2). The molecular mechanisms driving BM from breast cancer (B2BM) and other systemic cancers have been intensively studied in recent years (3). However, viable treatment strategies for clinical translation have been slow to emerge.

To identify molecular networks promoting B2BM, we conducted RNA-Seq on 4 isogenic cell line pairs, each consisting of parental and brain metastatic (BrM) derivatives (MDA-MB-231, HCC1954, BT474, and IBC3) (Supplemental Figure 1A; supplemental material available online with this article; https://doi.org/10.1172/jci.insight.193119DS1). Surprisingly, upregulated genes in BrM derivatives mapped to Gene Ontology and Reactome signatures associated with synaptic structure and transmission (Figure 1A and Supplemental Figure 1, B and C), with enriched pathways largely composed of critical synaptic components (Supplemental Figure 1D). Glutamatergic and GABAergic synapses represent the major excitatory and inhibitory connective frameworks, respectively, within the central nervous system (CNS). While recent data have implicated hijacking of both in primary glioma (4), the manner of their involvement and therapeutic tractability in BM remain uncertain.

To further characterize the synaptic components upregulated in B2BM, we assessed existing transcriptional data derived from 45 case-matched primary and BM breast cancer samples for glutamatergic (AMPA, NMDA, Kainate, and metabotropic) and GABAergic receptor subunits. We found that multiple components of each receptor type were significantly increased in B2BM (Figure 1, B and C, and Supplemental Figure 1, E–I). Moreover, using additional patient-derived datasets, we demonstrated that genes encoding several glutamatergic and GABAergic receptor subunits were upregulated specifically in B2BM compared to other common metastatic sites, namely bone and lung (Supplemental Figure 1, J–M, and Supplemental Figure 2, A–C). Finally, analyzing RNA-Seq data from patient-matched primary and lung and liver metastatic breast cancer samples (5) largely failed to demonstrate any significant increase in glutamatergic (Supplemental Figure 2, D–G, and Supplemental Figure 2, H–K) or GABAergic (Supplemental Figure 2L and Supplemental Figure 3, A–C) receptor components (except GABRD in lung metastasis). These results correlate with our isogenic cell line findings implicating glutamatergic and GABAergic synaptic complexes specifically in B2BM.

To functionally interrogate synaptic signaling in B2BM, we developed a murine model based on MDA-MB-231 BrM cells, initially derived from a patient with TNBC before repeated passaging in mice to extract a CNS-tropic clone. Intracardiac injection of MDA-MB-231 BrM cells in immunodeficient NSG mice consistently yielded BM within 4 weeks. We then subjected this model to treatment with either vehicle, the GABA receptor antagonist flumazenil, or levetiracetam, which inhibits glutamatergic synaptic transmission, starting 48 hours after MDA-MB-231 BrM intracardiac injection, and we began monitoring BM growth weekly by IVIS imaging. Strikingly, both treatment regimens significantly curbed BM as assessed by bioluminescence imaging (BLI) (Figure 1D and Supplemental Figure 3D), with levetiracetam significantly prolonging murine survival and with flumazenil showing an improved survival trend (Figure 1E). No similar reduction in lung metastasis was observed 4 weeks after intracardiac injection (Supplemental Figure 3E). While the potent in vivo efficacy of these compounds was not recapitulated in vitro, with no consistent dose response effect on viability seen in 4 parental/BrM cell line pairs (Supplemental Figure 4, A–H), MDA-MB-231-BrM cell treatment with glutamate significantly increased in vitro proliferation, with parallel GABA studies revealing a similar but nonsignificant trend (Supplemental Figure 4, I and J). Finally, metastatic tumor foci demonstrated elevated expression of both the AMPA receptor subunit GluR2 (Figure 1F) and the GABA receptor subunit GABA(A)Rγ-II (Figure 1G). Substantial staining for both receptor subunits was also seen in TNBC BMs, comparable with that of uninvolved brain regions in human samples (Figure 1, H and I). Taken together, these findings indicate that targeting glutamatergic and/or GABAergic synaptic transmission selectively impairs B2BM in vivo, likely by impacting the CNS cancer cell–neuron microenvironment.

In summary, we demonstrate a functional requirement for glutamatergic and/or GABAergic synaptic signaling in B2BM outgrowth. Recent literature has demonstrated the importance of electrically active synaptic structures—either direct neuron-to-cancer cell connections or tripartite complexes featuring pre- and postmitotic neurons and cancer cells—in the growth of primary gliomas and BM, respectively (6). Our work concurs with these findings, while also demonstrating that brain-tropic cancer cells proactively engage transcriptional programs for postsynaptic machinery. We also show, for the first time to our knowledge, that targeting glutamatergic and/or GABAergic synapses with existing therapeutics significantly impairs B2BM in vivo, informing clinical translation. Indeed, our findings suggest that low-dose levetiracetam and/or flumazenil could be proactively administered in patients with advanced breast cancer to reduce BrM outgrowth. Finally, we suspect that our results will not be limited to breast cancer, generalizing more broadly to other solid tumor types.

For detailed methods, information regarding sex as a biological variable, statistics, study approval, data availability, author contributions, and acknowledgments, see the Supplemental Methods.

## Supplementary Material

Supplemental data

Supporting data values

## Figures and Tables

**Figure 1:**
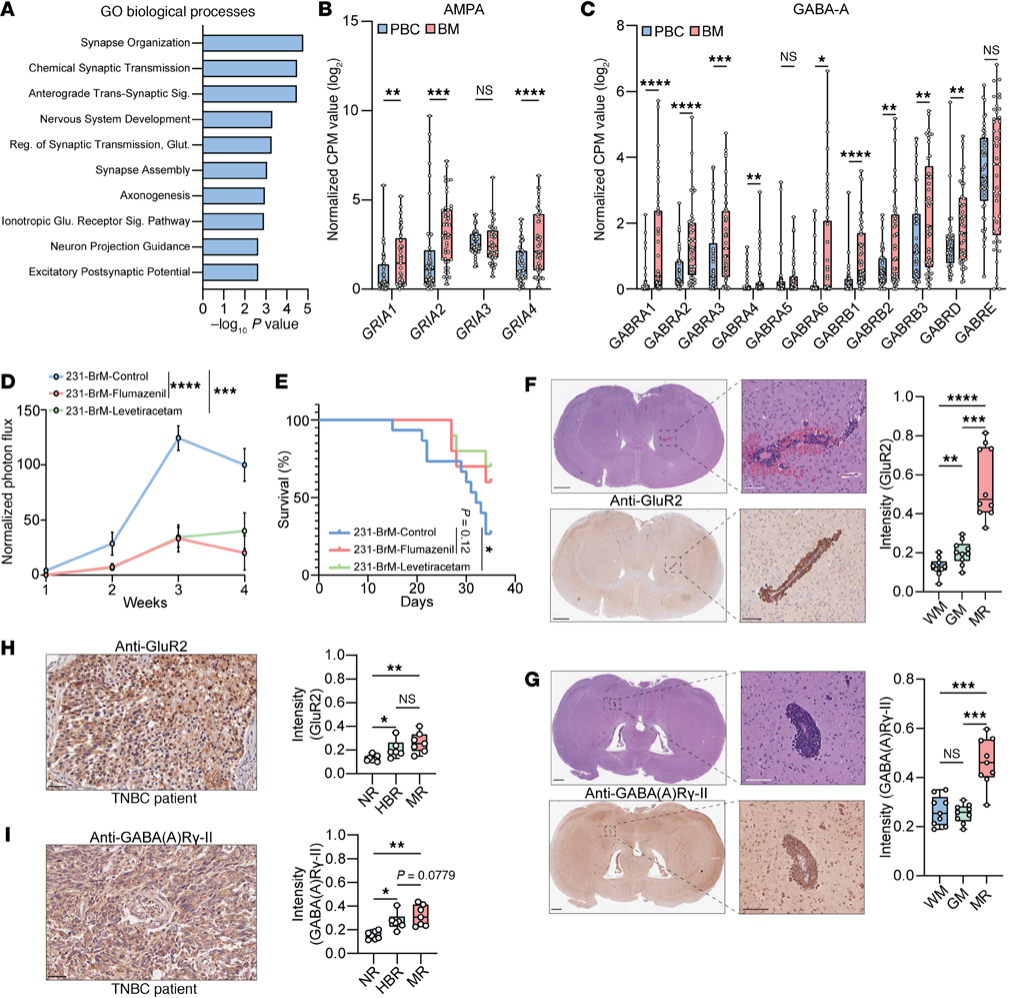
Synaptic transmission drives B2BM. (**A**) Box plots of Gene Ontology biological processes showing neuron-related transcriptional correlations for BrM cell lines relative to isogenic parentals (FC > 0.5 with *P* < 0.05). (**B** and **C**) Transcript levels of AMPA (**B**) and GABA-A (**C**) receptor components in primary breast cancer and B2BM samples (GSE184869) (*n* = 90); statistical analysis by Wilcoxon matched-pairs test. (**D**) Normalized photon flux from BLI images of control, flumazenil-, and levetiracetam-treated murine cohorts at the indicated time points after MDA-MB-231-BrM intracardiac injection; statistical analysis by multiple Mann-Whitney *U* test normalized to MDA-MB-231-Br3 control group at week 4. (**E**) Kaplan-Meier survival analysis for control and drug-treated murine cohorts following MDA-MB-231-BrM intracardiac injection until day 35 (vehicle [*n* = 15], flumazenil [*n* = 10], and levetiracetam [*n* = 10]). Statistical analysis by log rank (Mantel-Cox) test. (**F** and **G**) (Left) Representative H&E and corresponding GluR2 (**F**) or GABA(A)Rγ-II (**G**) IHC staining of NSG murine brain sections harboring MDA-231-BrM BMs. Scale bars: 500 μm (low power) and 100 μm (high power). (Right) Quantified GluR2 (**F**) or GABA(A)Rγ-II (**G**) IHC intensity in metastatic region (MR), white matter (WM), and gray matter (GM) regions [GluR2, *n* = 10; GABA(A)Rγ-II, *n* = 9]. Statistical analysis by 1-way ANOVA. (**H** and **I**) Representative IHC staining and intensity quantification for GluR2 (*n* = 7) (**H**) and GABA(A)Rγ-II (*n* = 7) (**I**) in human triple-negative B2BM tissue samples, assessing negative regions (NR), uninvolved healthy brain regions (HBR), and MR. Scale bars: 50 μm. Statistical analysis by 1-way ANOVA. Data are shown as mean ± SEM.

## Version 1 09/08/2025

In-Press Preview

## Version 2 10/08/2025

Electronic publication
