# Supplementary material for: Synaptic transmission promotes brain metastatic outgrowth in breast cancer
Source: JCI Insight. 2025 Sep 2;10(19):e193119. doi: 10.1172/jci.insight.193119 (PMC12513486; doi:10.1172/jci.insight.193119)
Supplement: Supplemental data [file jciinsight-10-193119-s227.pdf]

Synaptic transmission promotes brain metastatic outgrowth in breast cancer

Jayanta Mondal, Patrick Nylund, Prit Benny Malgulwar, William E. Johnson, Jason T. Huse

Supplementary Materials and Methods

Sex as a biological variable: This study exclusively examined female mice. Findings may not be entirely relevant to male mice.

In vivo animals experiment: 4-week-old female NOD.Cg-*Prkdc*^{scid} *Il2rg*^{tm1Wjl}/SzJ (NSG) mice were purchased from the Jackson Laboratory (Bar Harbor, ME, USA) for in vivo experiments.

Histology and immunohistochemistry: Formalin-fixed paraffin-embedded (FFPE) tissue sections of mouse or human brain were subjected to Immunohistochemistry (IHC) with hematoxylin counterstain. Briefly, brain tissue sections were dewaxed at 60°C overnight and then subjected to xylene followed by rehydration through alcohol gradients. Antigen retrieval was performed in 20 mM sodium citrate buffer (pH 6.0) in a microwave at 99°C for two 10-minute cycles. Following antigen retrieval, tissue sections were washed in ddH2O and then incubated in peroxidase blocking solution (12% MeOH, 3% H_2O_2, 85% ddH2O) for 30 minutes. Sections were then outlined using a hydrophobic pen and blocked with 2.5% normal goat serum (cat no. S-1000-20, Vector Laboratories, Newark, CA, USA) for 1 hour at room temperature. Blocking was followed by incubation with primary antibodies diluted in blocking buffer for 48 hours at 4°C. The primary antibodies used were GluR2 (1:25) (cat no. 32-0300, Invitrogen, Waltham, MA, USA) and GABA A Receptor Gamma 2 (GABA(A)Rγ-II) (1:25) (cat no. bs-4112R, BIOSSUSA, Boston, MA, USA). After primary antibody incubation, tissue sections were washed with PBS and PBST and then incubated with anti-rabbit secondary antibodies for 1 hour at room temperature. Immunohistochemistry was developed using the ImmPRESS® HRP Goat Anti-Rabbit IgG Polymer Detection Kit, Peroxidase (cat no. MP-7451, Vector Laboratories, Newark, CA, USA) for 7 minutes, followed by nuclear counterstaining with Hematoxylin. Visualization was further enhanced using bluing reagent (cat no. 67690001, Epridia, Kalamazoo, MI, USA). Slides were then dehydrated using alcohol gradients, immersed in xylene for 10 mins, and coverslipped. Brightfield images of stained slides were captured using a Keyence automated microscope. Immunohistochemistry analysis was performed in QuPath Version: 0.5.1. Positive staining was determined as intensity above average values of normal uninvolved brain, defined as grey matter (GM) or negative

cells. Negative cells in vivo were found in the murine corpus callosum, defined as white matter (WM) (anti-GluR2, in vivo n=10. One mouse was excluded as the section had no metastasis. Anti- GABA(A)Rγ-II, in vivo, n=9. Two mice were excluded as the section had unquantifiable metastatic burdens). Uninvolved "healthy brain regions" (HBR) were used as baseline and endothelial cells were used as negative regions (NR) in primary patient samples for GluR2 and GABA(A)Rγ-II protein expression. ROI was determined by H&E staining to identify infiltrating metastatic cells. (Anti-GluR2: n=7. One value was excluded as no uninvolved brain tissue was found). (Anti- GABA(A)Rγ-II: n=7. One value was excluded as no uninvolved brain tissue was found). The same ROI was then used for quantification after staining of consecutive tissue sections for GluR2 or GABA(A)Rγ-II. ROI in primary patient samples were identified by histopathological examination and quantified with QuPath Version: 0.5.1. Statistical analysis: One-way ANOVA.

Patient dataset analysis: Patient gene expression data was obtained from GSE184869, GSE110590, and GSE14017 and analyzed utilizing Biobase (2.58.0), GEOquery (2.66.0), limma (3.4.8.0), using Log2 fold change > 0.5 and adj p-value > 0.05 as thresholds. Variance analysis was done to determine significant probes in expression array analysis with tidyr (1.3.1) and dplyr (1.1.4). Average expression was conducted against multiple probes for each gene, where available, for array expression datasets. Samples missing values from the publicly available datasets, GSE184869, GSE110590, and GSE14017 were excluded and marked as NA. All data was analyzed and visualized in GraphPad Prism version 10.

Cell culture: MDA-MD-231, IBC3, HCC1954, BT474 and its BrM counterpart cell line were cultured in complete DMEM media (cat no. 10569010, Thermo Fisher Scientific, Waltham, MA, USA) , supplemented with 10% FBS (cat no. A5256701, Thermo Fisher Scientific, Waltham, MA, USA), and 1% Pen-strip (cat no. 30-002-CI, Thermo Fisher Scientific, Waltham, MA, USA) in biological triplicates. MDA-MD-231, HCC1954, and BT474 cell lines were graciously provided by Dr. Dihua Yu (University of Texas MD Anderson Cancer Center, Houston, TX). IBC3 cells were graciously provided by Dr. Bisrat Debeb (University of Texas MD Anderson Cancer Center, Houston, TX).

RNA isolation, RNA-seq and analysis: Total RNA samples were isolated using the Maxwell® RSC simplyRNA Cells Kit (cat no. AS1390, Promega, Madison, WI, USA) and submitted to the MDACC Advanced Technology

Genomics Core (ATGC) for processing and sequencing. Sample quality was assessed with Agilent BioAnalyzer (Agilent, Santa Clara, CA, USA) prior to sequencing. Libraries were prepared using the KAPA Stranded mRNA-Seq Kit (cat no. 50-196-5293, Thermo Fisher Scientific, Waltham, MA, USA) and sequenced on a NovaSeq6000 S1-200 flow cell (Illumina, San Diego, CA, USA) using the 100nt PE format. Raw fastq files were subjected to quality control (FASTQC) followed by hg38 alignment (RNASTAR version 2.7.8a). Raw transcript counts were determined using HTseq-count (version 0.9.1) and differentially expressed genes were determined with limma (version 3.4.8.0), followed by pathway and gene ontology analysis using EnrichR for upregulated genes with fold change > 0.5 and p value < 0.05. Correlated pathways and GOs obtained from EnrichR were plotted using GraphPad Prism. Neuron-related pathways have been depicted in the figures, while complete listed of pathways have been provide in relevant Source Data files.

In vivo drug treatment: 4-week-old NSG immunodeficient mice were randomly sorted into three groups (Vehicle (n=15), Flumazenil (cat no. 15-1788, MedChemExpress, Monmouth Junction, NJ, USA) (n=10) and Levetiracetam (n=10)) (cat no. 9001820, Cayman Chemical, Ann Arbor, Mi, USA) and injected with $2x10^5$ MDA-MB-231 BrM cells in the left ventricle on experimental day 0. From day 2 onwards, control mice were treated with DMSO, while experimental cohorts were treated with either 40 mg/kg of Flumazenil or 250 mg/kg of Levetiracetam through intraperitoneal route of injection every other day until day 28. Mice were IVIS imaged every week starting from day 7 and tracked for survival until day 35.

Bioluminescent imaging: Mice were anesthetized and intraperitoneally injected with 1.5 mg of D-luciferin at the indicated times. Animals were imaged in an IVIS 100 chamber within 5 min of D-luciferin injection, and data were recorded using Living Image software (IVIS Spectrum, Revvity, Waltham, MA, USA). To measure lung and brain metastasis, photon flux was calculated by drawing a region of interest using the Living Image (by PerkinElmer, Waltham, MA, USA) and Aura imaging (by Spectral Instruments imaging, Tucson, AZ, USA) software. Brain metastasis photon flux intensity for each mouse was normalized to the mean photon flux of the MDA-MB-231 control group at the 4 week-time point.

In vitro drug treatment: $6 x10^3$ cells of each line were seeded per well of a Greiner Bio-one cell culture 96 well, flat bottom, white, Cellstar® plate in complete DMEM media (cat no. 10569010, Thermo Fisher Scientific,

Waltham, MA, USA) containing 10% FBS (cat no. A5256701, Thermo Fisher Scientific, Waltham, MA, USA) and 1% Pen-Strep (cat no. 30-002-CI, Thermo Fisher Scientific, Waltham, MA, USA). After 24 hours, media were removed, and cells seeded in triplicate were treated with varying doses of Levetiracetam (cat no. 9001820, Cayman Chemical, Ann Arbor, Mi, USA) and Flumazenil (cat no. 15-1788, MedChemExpress, Monmouth Junction, NJ, USA) (0 uM, 0.1 uM, 0.625 uM, 4 uM, and 10 uM) prepared in DMEM (cat no. 10569010, Thermo Fisher Scientific, Waltham, MA, USA) containing 1% FBS (cat no. A5256701, Thermo Fisher Scientific, Waltham, MA, USA). 72 hours after drug treatment, viability was assessed by CellTiter-Glo® 2.0 Cell Viability Assay kit (cat no. G9242, Promega, Madison, WI, USA) using a Promega GloMax® Navigator Microplate Luminometer (Promega, Madison, WI, USA. All experiments were conducted in biological triplicates.

Proliferation Assay: Abcam Quick Cell Proliferation assay kit (cat no. ab65473, Abcam, Cambridge, UK) was used to measure proliferation of the MDA-MB-231-Parental and MDA-MB-231-Br3 brain metastatic cell lines upon treatment with L-glutamic Acid (Glu) (cat no. G8415, Sigma Aldrich, Saint Louis, MO, USA) or γ-Aminobutyric acid (GABA) (cat no. A2129 Sigma Aldrich, Saint Louis, MO, USA). In brief, 10,000 cells of each line were seeded per well of a Corning 96 well assay plate (Corning, 3610) in complete DMEM media (cat no. 10569010, Thermo Fisher Scientific, Waltham, MA, USA) containing 10% FBS (cat no. A5256701, Thermo Fisher Scientific, Waltham, MA, USA) and 1% Pen-Strep (cat no. 30-002-CI, Thermo Fisher Scientific, Waltham, MA, USA). After 24 hours, complete media were removed, replaced with low-serum media (containing 1% FBS), and cells seeded in triplicate were treated with 250 uM concentration of Glu or GABA. After 24 hours of treatment, 10 ul of WST-1/ECS solution (Abcam, Cambridge, UK) was added to each well. The cells were incubated for 2.5 hours in standard culture conditions, followed by shaking thoroughly for 1 min. Absorbance was subsequently measured using the Bio-tek uQuant plate reader at 450 nm wavelength. All experiments were conducted in biological triplicates.

Statistics and reproducibility: All statistical analyses not previously described were carried out using GraphPad Prism 10. No statistical method was used to predetermine sample size. For animal experiments, each mouse was counted as a biologically independent sample. Mice were randomized prior to commencing each in vivo experiment. Investigators were not blinded to allocation during the experimental procedures and during outcome

assessments. Testing for normality was done by collective assessment of D'Agostino-Pearson, Anderson-Darling, Shapiro-Wilk, and Kolmogorov-Smirnov tests. Statistical analyses used each figure are specified in the corresponding legends.

Murine Study approval: Mouse studies were conducted in accordance with protocols approved by the Institutional Animal Care and Use Committee of MD Anderson Cancer Center (00001597-RN02).

Human samples and ethics statement: De-identified brain metastasis tumor tissue derived from triple-negative breast cancer patients was obtained following informed consent from relevant patients, under the auspices of the front door tissue collection protocol previously approved by the University of Texas MD Anderson Cancer Center institutional review board (LAB03-0320).

Data Availability: All data are available in the Supporting data values file. RNA-seq fastq files have been deposited in the following repository E-MTAB-15278 at ArrayExpress. Request for further materials or other information will be addressed by the corresponding authors.

Funding Statement: J.M. is supported by the Cancer Prevention and Research Institute of Texas (CPRIT) Graduate Scholar Research Training Grant (RP210028) and the Schissler Foundation Fellowship. The use of the Small Animal Imaging Facility was supported by P30 CA016672 (MDACC) while the Advanced Technology Genomics core was also supported by NIH 1S10OD024977-01 (MDACC). This work was supported the MD Anderson Moonshot Program.

Acknowledgements: We would like to graciously acknowledge Dr. Erika Thompson and the MD Anderson Advanced Technology and Genomics Core (ATGC) for their technical support of this project. This work is the result of NIH funding, in whole or in part, and is subject to the NIH Public Access Policy. Through acceptance of this federal funding, the NIH has been given a right to make the work publicly available in PubMed Central.

Supplementary Figures & Legends

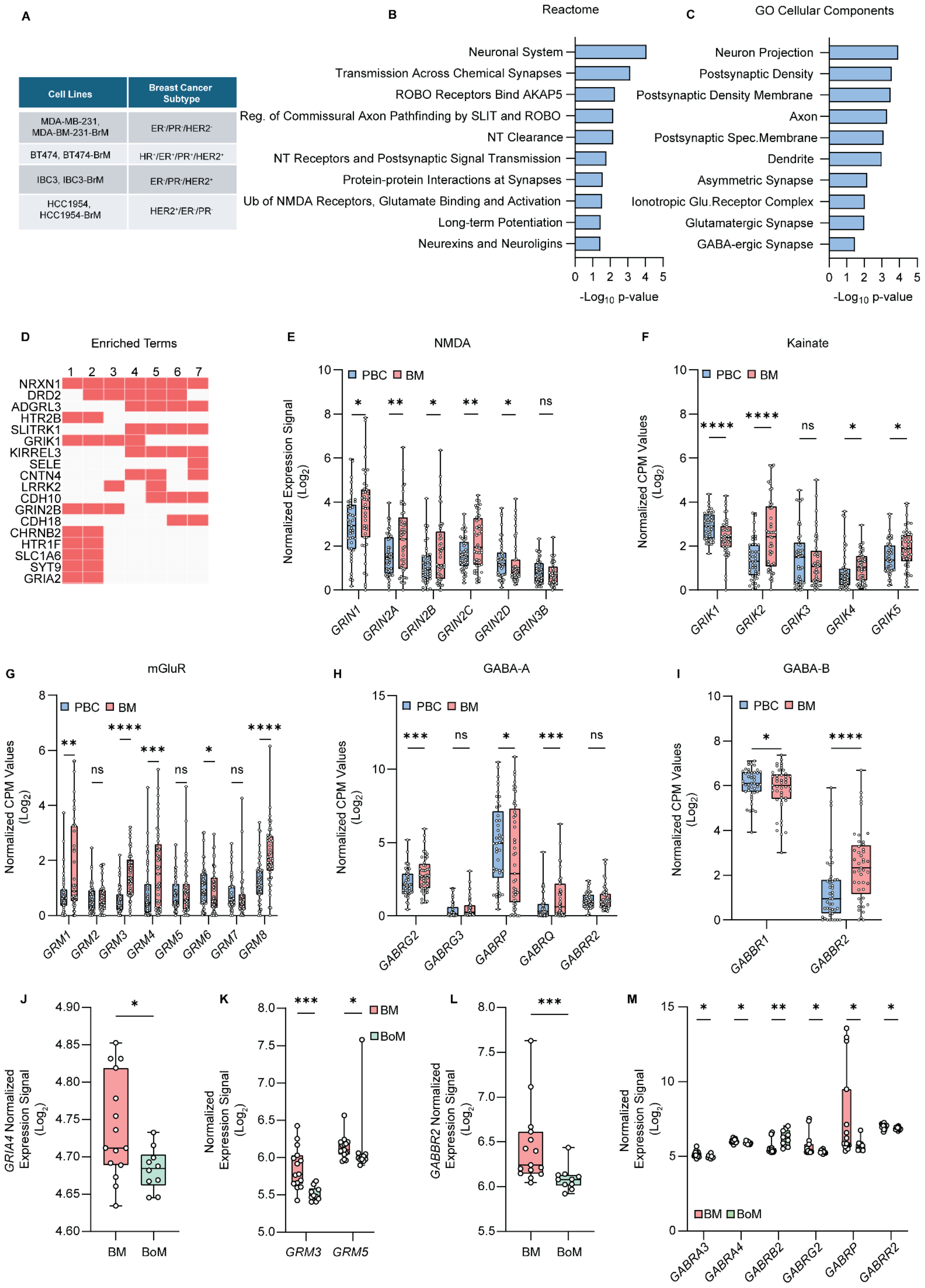
A
Cell Lines
Breast Cancer Subtype
MDA-MB-231, MDA-BM-231-BrM
ER-/PR-/HER2-
BT474, BT474-BrM
HR+/ER+/PR+/HER2+
IBC3, IBC3-BrM
ER-/PR-/HER2+
HCC1954, HCC1954-BrM
HER2+/ER-/PR-
B
Reactome
Neuronal System
Transmission Across Chemical Synapses
ROBO Receptors Bind AKAP5
Reg. of Commissural Axon Pathfinding by SLIT and ROBO
NT Clearance
NT Receptors and Postsynaptic Signal Transmission
Protein-protein Interactions at Synapses
Ub of NMDA Receptors, Glutamate Binding and Activation
Long-term Potentiation
Neurexins and Neuroligins
-Log10 p-value
C
GO Cellular Components
Neuron Projection
Postsynaptic Density
Postsynaptic Density Membrane
Axon
Postsynaptic Spec.Membrane
Dendrite
Asymmetric Synapse
Ionotropic Glu.Receptor Complex
Glutamatergic Synapse
GABA-ergic Synapse
-Log10 p-value
D
Enriched Terms
1 2 3 4 5 6 7
NRXN1
DRD2
ADGRL3
HTR2B
SLITRK1
GRIK1
KIRREL3
SELE
CNTN4
LRRK2
CDH10
GRIN2B
CDH18
CHRNB2
HTR1F
SLC1A6
SYT9
GRIA2
E
NMDA
PBC BM
Normalized Expression Signal (Log2)
GRIN1 GRIN2A GRIN2B GRIN2C GRIN2D GRIN3B
F
Kainate
PBC BM
Normalized CPM Values (Log2)
GRIK1 GRIK2 GRIK3 GRIK4 GRIK5
G
mGluR
PBC BM
Normalized CPM Values (Log2)
GRM1 GRM2 GRM3 GRM4 GRM5 GRM6 GRM7 GRM8
H
GABA-A
PBC BM
Normalized CPM Values (Log2)
GABRG2 GABRG3 GABRP GABRQ GABRR2
I
GABA-B
PBC BM
Normalized CPM Values (Log2)
GABBR1 GABBR2
J
GRIA4 Normalized Expression Signal (Log2)
BM BoM
K
Normalized Expression Signal (Log2)
BM
BoM
GRM3 GRM5
L
GABBR2 Normalized Expression Signal (Log2)
BM BoM
M
Normalized Expression Signal (Log2)
BM BoM
GABRA3 GABRA4 GABRB2 GABRG2 GABRP GABRR2

Supplementary Figure 1. Transcripts of synaptic components are selectively upregulated in BM. (A) Tabulated features of isogenic human-derived BrM and parental cell lines subjected to RNA-seq. **(B)** Reactome pathways enriched in BrM cell line derivatives relative to isogenic parental cell lines; box plot showing enriched pathways obtained from differentially expressed upregulated genes with (FC>0.5) with p value<0.05. **(C)** GO analysis for cellular components showing enhanced transcriptional correlations for BrM cell line derivatives relative to isogenic parental cell lines; box plots showing enriched pathways obtained from differentially expressed upregulated genes with (FC>0.5) with p value<0.05. RNA-seq from each BrM and parental cell line was conducted in three biological replicates (n=3). **(D)** Clustergram of GO biological processes genes upregulated in BrM cell line derivatives relative to isogenic parental cell lines. (Enriched terms: 1. Anterograde Trans-Synaptic Signaling (GO:0098916); 2. Chemical Synaptic Transmission (GO:0007268); 3. Regulation of Synaptic Transmission, Glutamatergic (GO:0051966); 4. Nervous System Development (GO:0007399); 5. Synapse Organization (GO:0050808); 6. Cell Junction Assembly (GO:0034329); 7. Cell-Cell Adhesion Via Plasma-Membrane Adhesion Molecules (GO:0098742)) **(E-I)** Gene expression data for major glutamatergic receptor (**(E)** NMDA, **(F)** Kainate **(G)** mGluR) and GABAergic receptor (**(H)** GABA-A and **(I)** GABA-B) components derived from a 45-patient dataset of matched primary breast cancer and B2BM samples (GSE184869) (n=90). Statistical analysis performed using matched Multiple Wilcoxon test. **(J-M)** Selected comparisons of normalized expression levels of glutamatergic (**(J)** *GRIA4*, **(K)** *GRM3* and *GRM5*) and GABAergic (**(L)** *GABBR2*, **(M)** *GABRA3*, *GABRA4*, *GABRB2*, *GABRP* and *GABRR2*) receptor component transcripts for breast cancer brain metastasis vs bone metastasis samples in the GSE14017 patient cohort (n_{brain}=15, n_{lung}=4 and n_{bone}=10). Statistical analysis performed using Mann-Whitney test. All values are presented as standard error of the mean. *p < 0.05, **p < 0.01, ***p < 0.001, ****p < 0.0001.

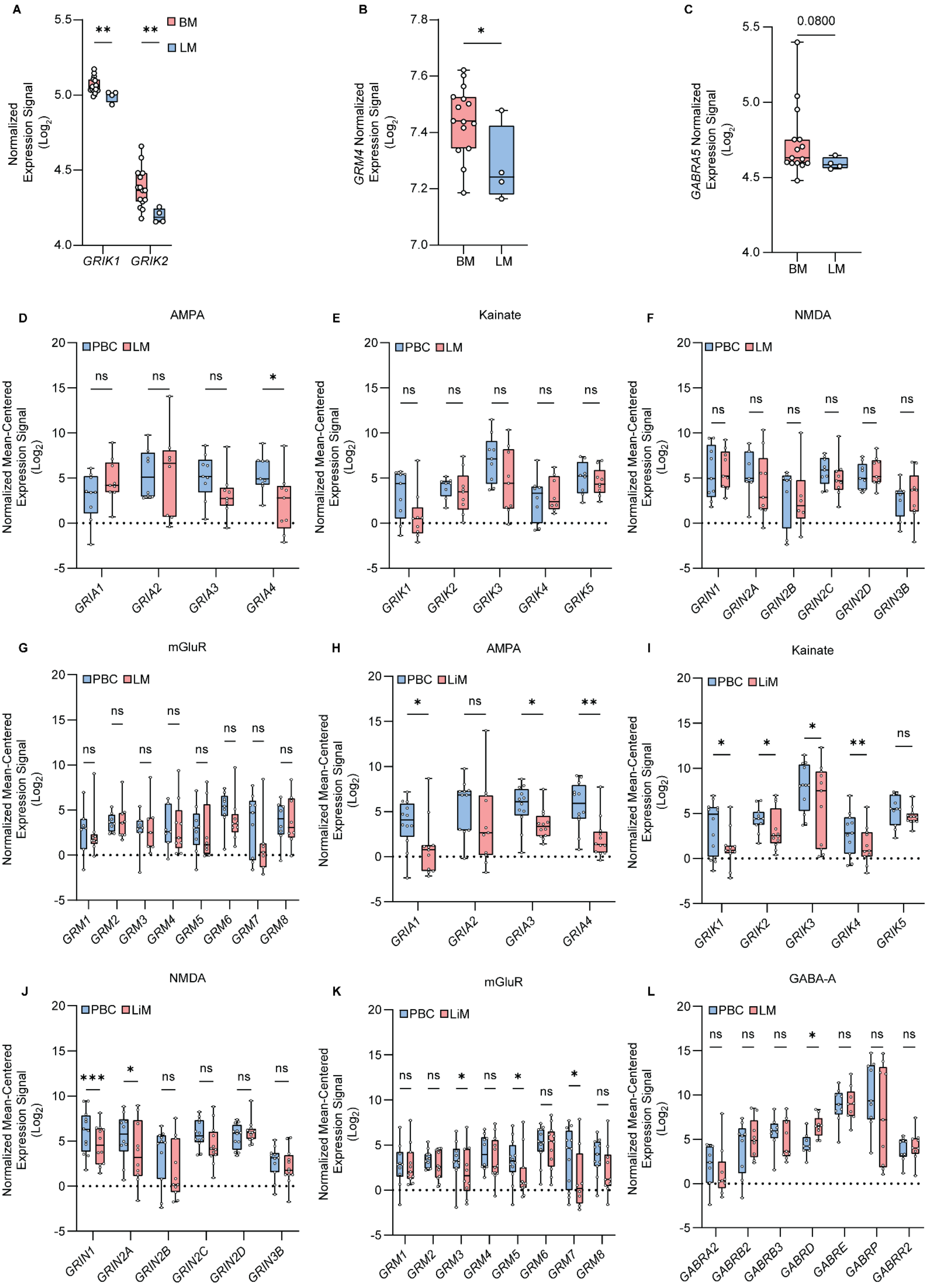
A
Normalized Expression Signal (Log2)
5.5
5.0
4.5
4.0
**
**
BM
LM
GRIK1
GRIK2
B
GRM4 Normalized Expression Signal (Log2)
7.8
7.6
7.4
7.2
7.0
*
BM
LM
C
GABRA5 Normalized Expression Signal (Log2)
5.5
5.0
4.5
4.0
0.0800
BM
LM
D
AMPA
PBC
LM
Normalized Mean-Centered Expression Signal (Log2)
20
15
10
5
0
-5
ns
ns
ns
*
GRIA1
GRIA2
GRIA3
GRIA4
E
Kainate
PBC
LM
ns
ns
ns
ns
ns
GRIK1
GRIK2
GRIK3
GRIK4
GRIK5
F
NMDA
PBC
LM
ns
ns
ns
ns
ns
ns
GRIN1
GRIN2A
GRIN2B
GRIN2C
GRIN2D
GRIN3B
G
mGluR
PBC
LM
ns
ns
ns
ns
ns
ns
ns
ns
GRM1
GRM2
GRM3
GRM4
GRM5
GRM6
GRM7
GRM8
H
AMPA
PBC
LiM
*
ns
*
**
GRIA1
GRIA2
GRIA3
GRIA4
I
Kainate
PBC
LiM
*
*
*
**
ns
GRIK1
GRIK2
GRIK3
GRIK4
GRIK5
J
NMDA
PBC
LiM
***
*
ns
ns
ns
ns
GRIN1
GRIN2A
GRIN2B
GRIN2C
GRIN2D
GRIN3B
K
mGluR
PBC
LiM
ns
ns
*
ns
*
ns
*
ns
GRM1
GRM2
GRM3
GRM4
GRM5
GRM6
GRM7
GRM8
L
GABA-A
PBC
LM
ns
ns
ns
*
ns
ns
ns
GABRA2
GABRB2
GABRB3
GABRD
GABRE
GABRP
GABRR2

Supplementary Figure 2. Transcripts of synaptic components are not similarly upregulated in non-CNS breast cancer metastasis. **(A-C)** normalized expression levels of **(A)** *GRIK1* and *GRIK2*, **(B)** *GRM4*, and **(C)** and *GABRA5* from metastasis samples from the GSE14017 patient cohort (n_{brain}=15, n_{lung}=4 and n_{bone}=10). Statistical significance determined by multiple Mann-Whitney test or Kruskal-Wallis test. **(D-G)** Gene expression data for major glutamatergic receptor (**(D)** AMPA, **(E)** Kainate, (**F**) NMDA and **(G)** mGluR components derived from a patient dataset of matched primary breast cancer and breast to lung metastasis samples (GSE110590) (n=18). Statistical analysis performed using Multiple paired t-test. (**H-K**) Plotted gene expression data for major glutamatergic receptor ((**H**) AMPA, (**I**) Kainate and (**J**) NMDA) and (**K**) mGluR) components derived from a patient dataset of matched primary breast cancer and breast to liver metastasis samples (GSE110590) (n=24). (**L**) Gene expression data for GABAergic receptor GABA-A components derived from a patient dataset of matched primary breast cancer and breast to lung metastasis samples (GSE110590) (n=18). Statistical analysis performed using matched Multiple Wilcoxon test. All values are presented as standard error of the mean. *p < 0.05, **p < 0.01, ***p < 0.001, ****p < 0.0001.

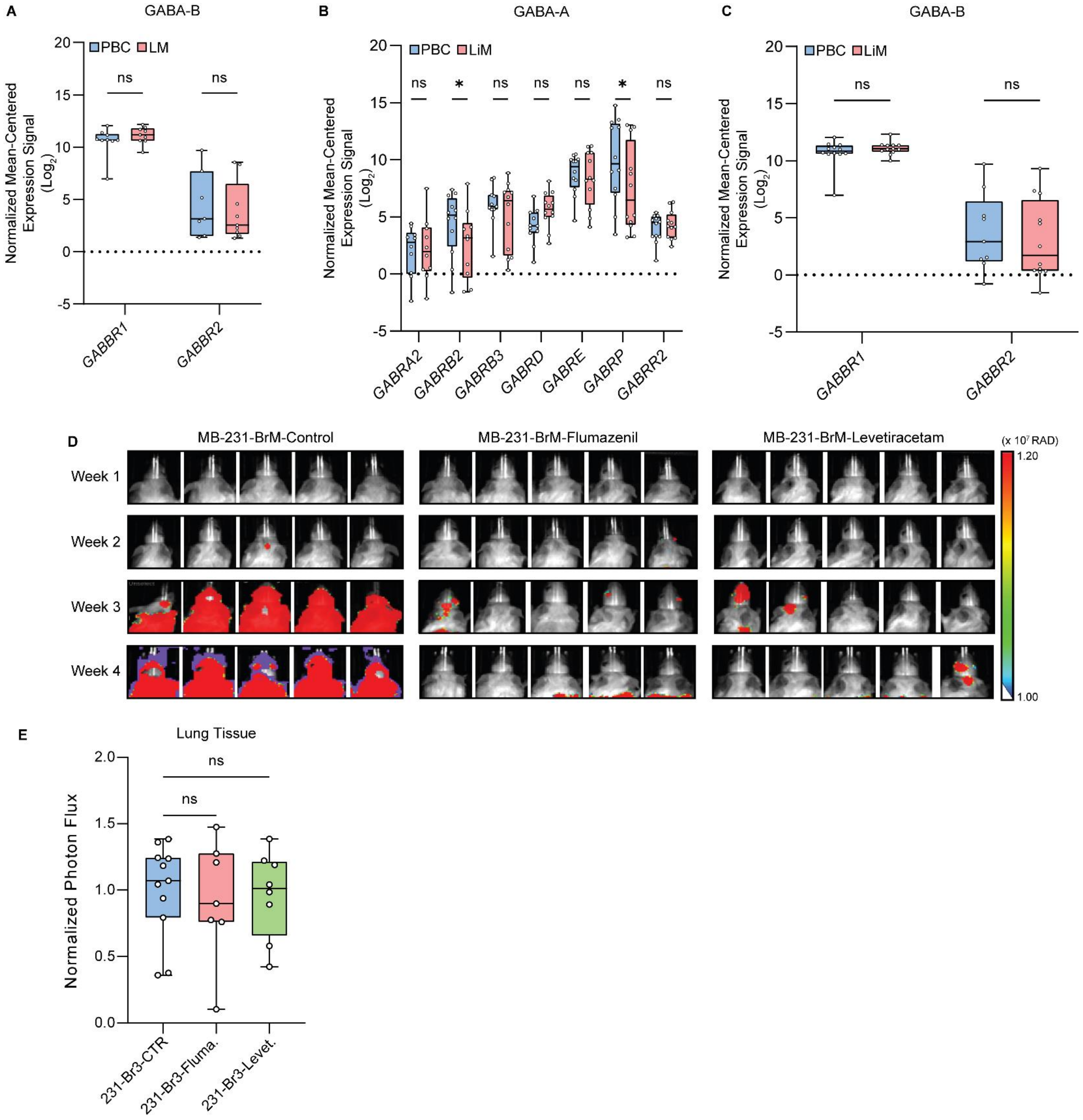

Supplementary Figure 3. Liver metastasis does not show increased expression of synaptic components while targeting glutamatergic and/or GABAergic synaptic transmission selectively impairs B2BM in vivo. (**A**) data for GABAergic receptor GABA-B components derived from a patient dataset of matched primary breast cancer and breast to lung metastasis samples (GSE110590) (n=18). (**B-C**) Gene expression data for GABAergic receptor ((**B**) GABA-A and (**C**) GABA-B) components derived from a patient dataset of matched primary breast

cancer and breast to liver metastasis samples (GSE110590) (n=24). Statistical analysis performed: (**A-B**) Multiple paired t-test and (**C**) Matched Multiple Wilcoxon test. (**D**) Representative BLI imaging of the head regions of female NOD.Cg-*Prkdc*^{scid} *Il2rg*^{tm1Wjl}/SzJ (NSG) mice 1-4 weeks following intra-cardiac injection with MDA-MB-231-BrM cells; control and treated cohorts are indicated. Scaling normalization was done by reference-based normalization against week 4 control samples, thus generating a unified scale for entire experiment. (**E**) Plot comparing breast cancer lung metastatic burden (as inferred by regional normalized photon flux) in the indicated murine cohorts 4 weeks after MDA-MB-231-BrM cell intra-cardiac injection. 231-Br3-CTR was used for reference-based normalization (Vehicle (n=15), Flumazenil (n=10) and Levetiracetam (n=10)). Statistical analysis performed by One-way ANOVA. *$p < 0.05$, **$p < 0.01$, ***$p < 0.001$, ****$p < 0.0001$.

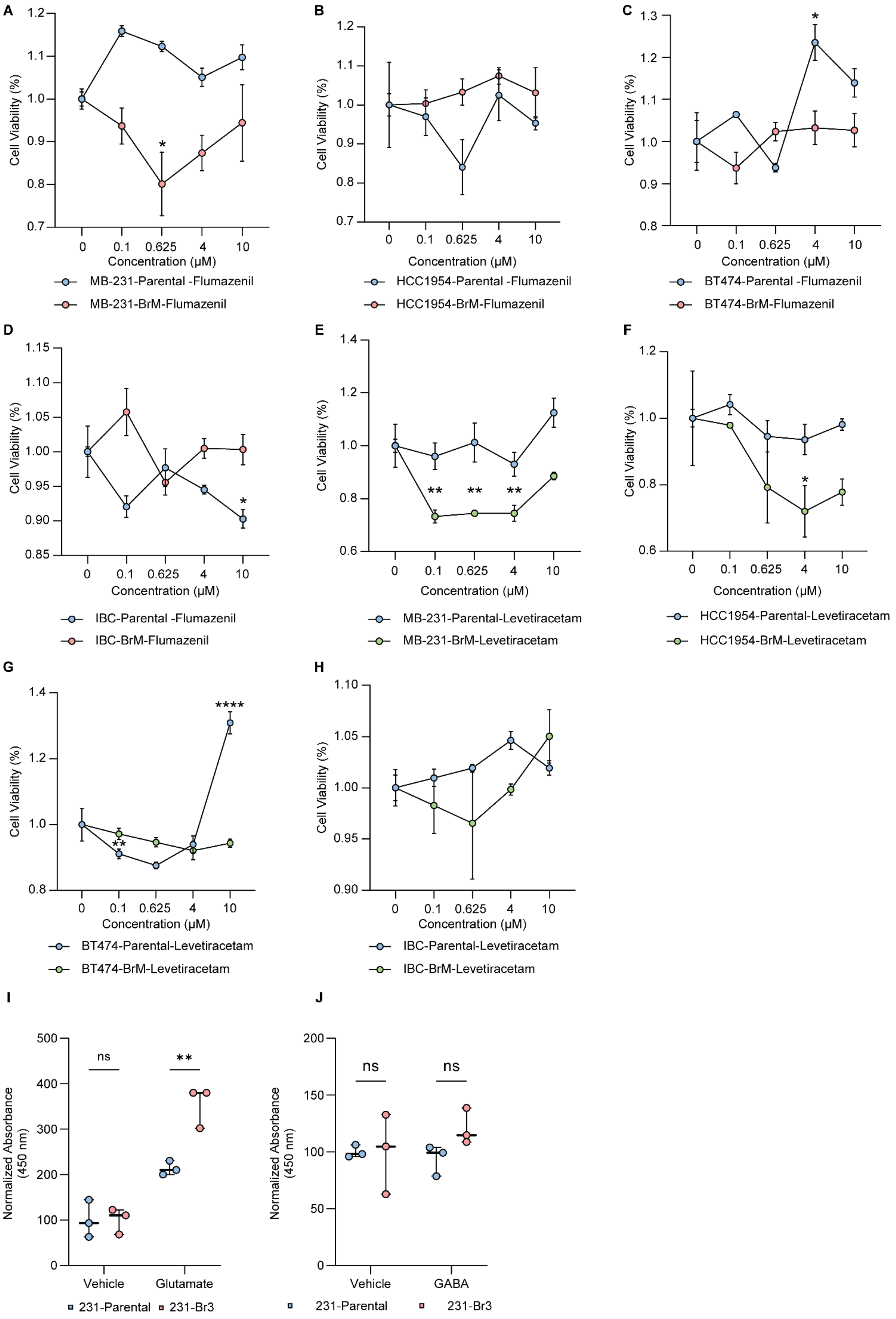
A
Cell Viability (%)
Concentration (μM)
MB-231-Parental -Flumazenil
MB-231-BrM-Flumazenil
B
HCC1954-Parental -Flumazenil
HCC1954-BrM-Flumazenil
C
BT474-Parental -Flumazenil
BT474-BrM-Flumazenil
D
IBC-Parental -Flumazenil
IBC-BrM-Flumazenil
E
MB-231-Parental-Levetiracetam
MB-231-BrM-Levetiracetam
F
HCC1954-Parental-Levetiracetam
HCC1954-BrM-Levetiracetam
G
BT474-Parental-Levetiracetam
BT474-BrM-Levetiracetam
H
IBC-Parental-Levetiracetam
IBC-BrM-Levetiracetam
0
0.1
0.625
4
10
I
Normalized Absorbance (450 nm)
ns
**
Vehicle
Glutamate
231-Parental
231-Br3
J
ns
ns
Vehicle
GABA
231-Parental
231-Br3

Supplementary Figure 4. Glutamate treatment significantly increases proliferation of breast cancer brain metastatic cells compared to parental counterparts in vitro. (A-H) Cell viability assays for case-matched human-derived BrM and parental cell lines subjected to increasing doses of either Flumazenil (A-D) or Levetiracetam (E-H) in vitro for 72 hours. All experiments were performed in biological triplicates (n=3). Statistical analysis was performed using two-way ANOVA. All comparisons were conducted between the different concentrations in each cell line compared to vehicle. Asterisks indicate significant differences while absence indicates non-significance. All values are presented as standard error of the mean. (**I-J**) Absorbance plot of MDA-MB-Parental and MDA-MB-231-BrM cell lines treated with 250 uM of L-glutamic acid (**I**) or GABA (**J**) for 24 hours. All experiments were performed in biological triplicates (n=3). Statistical analysis performed using two-way ANOVA. All values are presented as standard error of the mean. *$p < 0.05$, **$p < 0.01$, ***$p < 0.001$, ****$p < 0.0001$.
